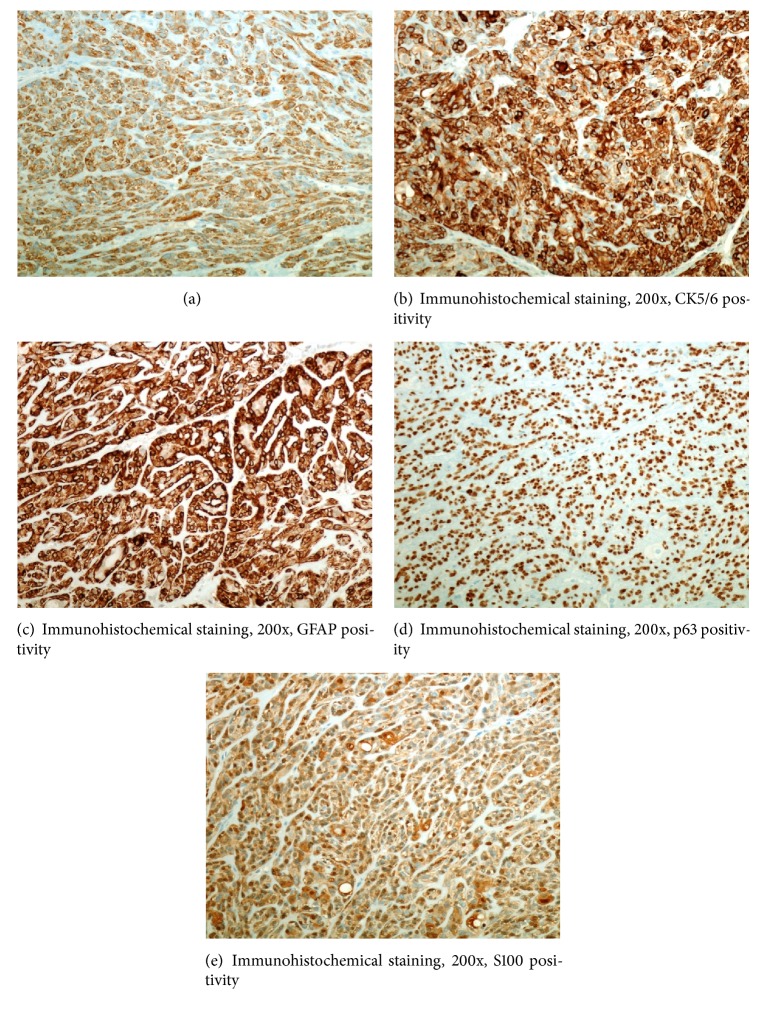# Myoepithelioma of the Parotid Gland: A Case Report with Review of the Literature and Classic Histopathology

**DOI:** 10.1155/2017/6036179

**Published:** 2017-08-16

**Authors:** Mark Weitzel, Jason E. Cohn, Harvey Spector

**Affiliations:** ^1^Department of Otolaryngology-Head and Neck Surgery, Philadelphia College of Osteopathic Medicine, 4190 City Line Avenue, Philadelphia, PA, USA; ^2^Department of Pathology, Crozer Chester Medical Center, One Medical Center Boulevard, Upland, PA, USA

## Abstract

Myoepithelioma is a rare salivary gland neoplasm. They most commonly affect the major and minor salivary glands with the parotid gland being the most common, approximately 40%. Only 1% of all salivary gland neoplasms are myoepitheliomas. Myoepithelioma is usually a benign tumor arising from neoplastic myoepithelial or basket cells which are found between the basement membrane and the basal plasma membrane of acinar cells. They also contain multiple cellular elements. We present a case of a 73-year-old female with myoepithelioma of the parotid gland, an extremely rare neoplasm. There have been approximately 42 cases reported through 1985 and fewer than 100 cases through 1993. We will discuss the clinical presentation, pathophysiology, diagnosis, and treatment of such neoplasms.

## 1. Introduction

Of all benign major and minor salivary gland neoplasms, myoepitheliomas account for 2.2% and 5.7%, respectively. The parotid gland is affected in approximately 40% of cases. In total, myoepitheliomas only account for 1% of all salivary gland neoplasms. The overwhelming majority of myoepitheliomas are benign but malignant transformation can take place in recurrent cases and cases left untreated [1–3]. We present the first known case, to the best of our knowledge, at our institution of myoepithelioma occurring in the left parotid gland.

## 2. Case Report

A 73-year-old female with a past medical history of hypertension, peripheral arterial disease, asthma, gastroesophageal reflux disease, and diabetes mellitus presented to our otolaryngology clinic with the complaint of a “nodule” behind her left ear. She stated that the mass had been slowly increasing in size over the last 5 years. It was described as nonpainful and she had never experienced any discharge from the area. Her only other complaints were decreased hearing, xerostomia, and hoarseness; no dysphagia or weight loss was reported. Past surgical history was significant for angioplasty and stent placement in her legs. Social history was significant for a 20-pack-year tobacco history. No significant family history was reported. On physical examination pertinent findings consisted of a left posterior auricular mass approximately 2 centimeters (cm) that was fixed and nontender. All cranial nerves, most notably the facial nerve, were intact. No cervical adenopathy was palpated. Nasopharyngeal laryngoscopy demonstrated arytenoid edema consistent with reflux but the true vocal folds were mobile and no other lesions were visualized. Treatment plan at that time consisted of smoking cessation and a computed tomography (CT) scan with contrast of the neck.

The CT scan revealed a multilobulated and cystic mass in the left parotid gland with an infiltrating appearance consistent with neoplasm. There was no pathologic cervical lymphadenopathy reported. The differential diagnosis at this time included benign mixed tumor, Warthin's tumor, epidermoid tumor, and adenoid cystic carcinoma. A fine needle aspiration was performed at this time and the results were nondiagnostic. The decision was made to proceed with a left parotidectomy.

**Figure 1:**
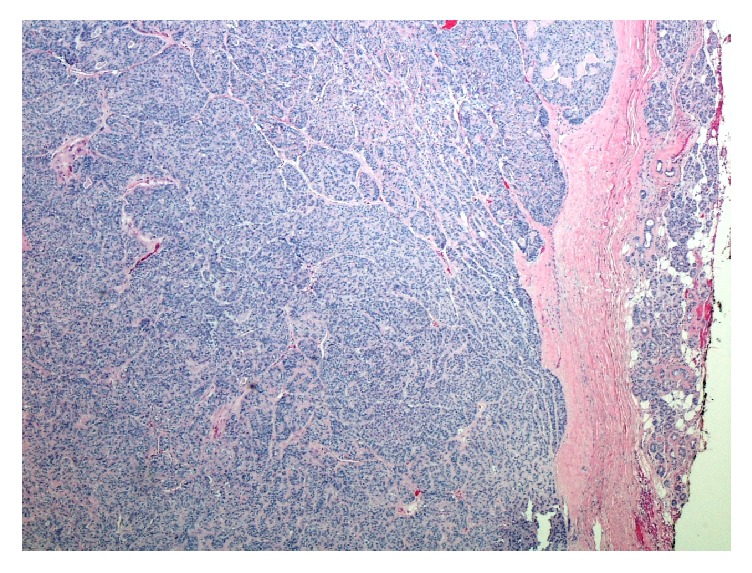
Hematoxylin and eosin, 40x, myoepithelial cells arranged in a fascicular nested pattern separated by collagen stroma.

**Figure 2:**
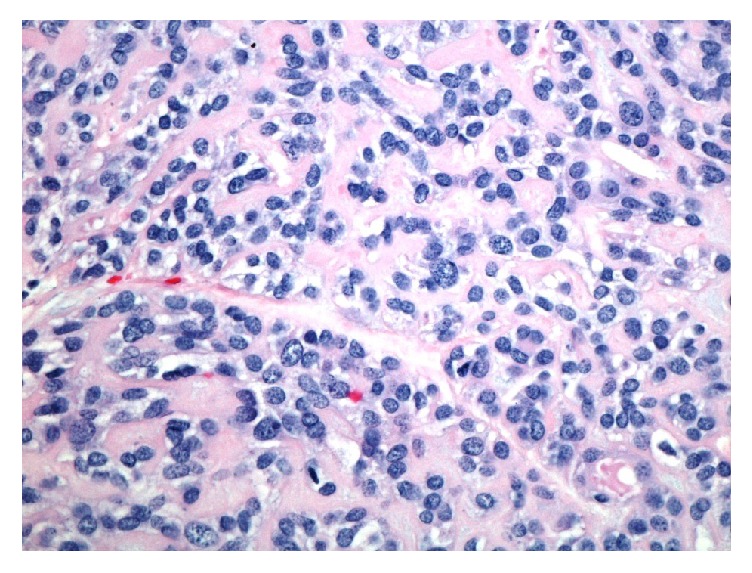
Hematoxylin and eosin, 400x, Tumor showing an epithelioid pattern of uniform cells with central small nuclei with fine chromatin, inconspicuous nucleoli, and an eosinophilic cytoplasm. There is a mild degree of nuclear atypia without mitosis.

Left superficial parotidectomy with facial nerve monitoring was performed approximately one month after presentation. A 2 × 2 cm lesion was removed from the tail of the left parotid gland. The gross description was an encapsulated, lobulated mass with a brown-yellowish coloration. The specimen was sent for analysis by the pathology department. The tumor was composed of neoplastic myoepithelial cells arranged in a fascicular nested pattern separated by collagen stroma (Figure 1). There was an epithelioid pattern of uniform cells with central small nuclei with fine chromatin, inconspicuous nucleoli, and an eosinophilic cytoplasm. Mild degree of nuclear atypia without mitosis was noted (Figure 2). Immunohistochemical staining of the specimen was positive for calponin (Figure 3(a)), CK5/6 (Figure 3(b)), GFAP (Figure 3(c)), p63 (Figure 3(d)), S100 (Figure 3(e)), CK7, vimentin, and SMA. CK 20 was negative. No malignant features were identified and the diagnosis of epithelioid myoepithelioma was made.

The patient recovered from the surgery without complications and her facial nerve was functioning well. The patient had no sign of recurrence at 10 months and is currently being seen regularly for routine monitoring.

## 3. Discussion

The description of a salivary neoplasm resembling a myoepithelioma was first attempted in 1943 [4]. Thereafter, this entity was referred to as several other neoplasms including “parotid clear cell adenoma of possible myoepithelial origin” and “adenomyoepithelioma” [5]. Myoepithelioma of the salivary gland was first officially recognized as a subtype of salivary neoplasms in 1991 [6]. It is a rare benign mass arising from neoplastic myoepithelial or basket cells, which are found between the basement membrane and the basal plasma membrane of acinar cells. They are made up of numerous cellular elements including smooth muscle actin, myosin, and intermediate filaments. Myoepithelial cells are thought to have contractile units that aid in excreting glandular secretions [1, 2].

Myoepitheliomas only account for approximately 1% of all salivary neoplasms [3]. Primarily they will affect the parotid gland (~40%) and minor salivary gland sites (~21%) [7–10]. There have been rare instances of submandibular gland myoepitheliomas [7, 8]. Neoplastic myoepithelial cell tumors can be found in nearly all exocrine gland tissues such as skin, soft tissue, sweat glands, breast, lacrimal glands, Bartholin's glands, nasal septum, nasopharynx, larynx, trachea, lung, esophagus, retroperitoneum, and prostate gland. One exception would be the pancreas [5, 11–13]. A comprehensive differential diagnosis would include abscess, mucocele, schwannoma, neurofibroma, leiomyoma, benign fibrous histiocytoma, extramedullary plasmacytoma, rhabdomyosarcoma, smooth muscle neoplasms, pleomorphic adenoma, mucoepidermoid carcinoma, myoepithelial carcinoma, and other benign and malignant salivary gland neoplasms [14]. In particular, our case appeared to be cystic in nature. Myoepithelioma tumors have also been misdiagnosed as a parotid cyst [15].

Of all benign major and minor salivary gland neoplasms, myoepitheliomas account for 2.2 and 5.7%, respectively. On gross inspection, they have a solid, tan or yellow-tan, glistening cut surface, similar to what was seen in our gross examination. Subtypes of myoepitheliomas are classified by cell morphology: spindle (interlacing fascicles with a stroma-like appearance), plasmacytoid/hyaline (polygonal cells with eccentric nuclei and dense, nongranular or hyaline, abundant eosinophilic cytoplasm), epithelioid (nests or cords of round to polygonal cells, with centrally located nuclei and a variable amount of eosinophilic cytoplasm), and clear (polygonal cells with abundant optically clear cytoplasm, containing large amounts of glycogen but missing mucin or fat) [4]. Of those subtypes, spindle cell type is most common (65%) followed by plasmacytoid (20%) [7]. Due to histological and cytogenetic similarities, a myoepithelioma can sometimes be misdiagnosed as a pleomorphic adenoma. The most common and shared cytogenic abnormality is seen on chromosome 12q. Immunohistochemical analysis can aid in the diagnosis with immunoreactivity to S-100, cytokeratin (specifically 7 and 14), p63, GFAP, calponin, and myogenic markers (actin, myosin) [4, 7, 11]. These immunohistochemical features were found in our specimen.

Most of these tumors occur in adults, with some cases reported in the pediatric population. Both genders are affected equally. The average age of a patient with myoepithelioma is 44 years, with a range of 9–85 years. The most common clinical presentation is a slow growing, painless mass, as seen in the clinical presentation of our patient [4]. They usually grow locally without invading surrounding structures, such as the facial nerve which is unlike other benign parotid masses [5]. The diagnosis is usually made with a combination of radiologic imaging and tissue histology. In one study using CT scans, the majority of cases showed well circumscribed, smooth or lobulated, homogenous enhancing lesions, similar to what was seen in our case [16]. Typical MRI studies show a well-defined homogenous isointense and hyperintense mass on T1- and T2-weighted imaging, respectively [6]. The treatment of choice is surgical excision. However, the role of chemoradiation is not well established due to the rarity of this neoplasm [5, 8].

The recurrence rate for myoepitheliomas has been reported to be 15–18%, with possible malignant transformation in long-standing tumors or recurring disease [4]. However, one study demonstrated that only 1 out of 16 myoepitheliomas recurred over a 7-year period [17]. Malignant transformation has been attributed to the overexpression of c-kit receptors and p53 mutations. Myoepithelial carcinoma can also arise de novo. Carcinoma only comprises approximately 10% of all myoepithelial neoplasms [8]. Some characteristics of a carcinoma include aggressive behavior (infiltrative and destructive growth), increased mitotic activity, necrosis, lack of myofilaments (therefore more monomorphic than benign myoepithelioma), and cell pleomorphism [2, 4].

After complete surgical excision, routine follow-up is needed in the treatment algorithm of myoepitheliomas secondary to the recurrence rate. Currently in our patient there has not been any sign of recurrence. In conclusion, although myoepithelioma of the parotid gland is the most common site for a myoepithelioma to appear they are still a rare salivary gland neoplasm. It is challenging to obtain an exact number of cases that have been reported in the literature. However, it has been elucidated that there were 42 cases reported through 1985 and fewer than 100 cases through 1993 [18]. Additionally, there has never been myoepithelioma of any salivary gland at our affiliated institutions. It is possible that the occurrence rate is higher but that some are misdiagnosed as other salivary gland neoplasms such as pleomorphic adenoma or a parotid cyst. It is important to use proper immunohistochemical staining in such cases when myoepithelioma is suspected so that proper treatment and follow-up can be implemented.

## Figures and Tables

**Figure 3:**